# Second-generation cryoballoon ablation for recurrent atrial fibrillation after an index cryoballoon procedure: a staged strategy with variable balloon size

**DOI:** 10.1007/s10840-018-0418-z

**Published:** 2018-08-08

**Authors:** Sjoerd W. Westra, Stijn P. G. van Vugt, Sümeyye Sezer, Reinder Evertz, Martin E. Hemels, Rypko J. Beukema, Carlo de Asmundis, Marc A. Brouwer, Gian-Battista Chierchia

**Affiliations:** 1 Department of Cardiology, Radboud University Medical Center, Geert Grooteplein Zuid 10, 6525 GA Nijmegen, The Netherlands; 2 Heart Rhythm Management Center, Postgraduate Course in Cardiac EP and pacing, Universitair Ziekenhuis Brussels, Laarbeeklaan 101, 1090 Jette, Brussels, Belgium

**Keywords:** Atrial fibrillation, Pulmonary vein isolation, Cryoballoon ablation, Redo procedure

## Abstract

**Purpose:**

Currently, information on the optimal approach of redo procedures for paroxysmal atrial fibrillation (PAF) is limited. Radiofrequency ablation is the preferred technique, with reported success rates of 50–70% at 1–2 years, whereas only few reports exist on redo cryoballoon (CB) ablations. We describe outcomes on a systematic approach of repeat procedures with a second-generation cryoballoon (CB-2) after a successful index CB ablation.

**Methods:**

Cohort study of 40 consecutive patients with recurrent PAF (55% male), median CHA_2_DS_2_-VASc score 1 (IQR 0–3). Per protocol, a staged variable balloon size strategy was followed with a different balloon size during the redo as compared to the index procedure. Minimal follow-up was 12 months (median 17 months [IQR 14–39]).

**Results:**

Overall, 120 pulmonary veins (PVs) (75%) showed chronic isolation: 64% (41/64) for first-generation cryoballoon (CB-1) and 82% (79/96) for CB-2 index procedures, respectively (*p* = 0.01). The overall mean number of reconnected PVs per patient was 1.0 (40/40): 1.4 for CB-1 and 0.7 for CB-2 index procedures (*p* = 0.008). Phrenic nerve palsies (*n* = 7) resolved before the end of the procedure. At 1 year, 70% of patients were free of recurrent AF. In multivariate analysis, the only independent predictor of recurrence was the number of prior cardioversions.

**Conclusions:**

A systematic approach of repeat procedures with a CB-2 using a different balloon size than during the index CB ablation is safe, with acceptable 1-year outcomes. Future comparative studies on the optimal redo technique and approach are warranted to further improve rhythm control in AF.

## Introduction

Over the years, pulmonary vein isolation (PVI) has become an accepted therapy for drug-refractory paroxysmal atrial fibrillation (PAF) [1, 2]. One-year success rates are about 70%, and up to 20% of patients require a redo procedure [3–6]. At present, little information is available on the optimal energy source and approach during repeat procedures.

The vast majority of data pertains to radiofrequency (RF) ablation, and success rates vary from 80 to 90% at 4–6 months to 58–70% at 12–24 months [1, 2, 7–11]. As of yet, experiences with cryoballoon (CB) ablation as a redo procedure after an initial RF ablation are limited [11, 12]. With regard to a redo CB ablation after a successful index cryoablation, only one report has been published [13]. In that cohort, the first-generation CB (CB-1) was used with a success rate of 60% 1 year after the redo. Currently, the second-generation CB (CB-2) is used, known for its enhanced surface cooling due to the double amount of nitrogen jets [14]. Notably, a recent paper on a series of redo procedures demonstrated significantly lower numbers of reconnected pulmonary veins in patients with a prior CB-2 procedure than in patients with a prior RF ablation [7].

With regard to the preferred approach in the redo setting, the evidence for ablation on top of PVI versus PVI alone is not uniform, and randomized data is scarce [1, 15]. In this context, more information on redo procedures is warranted, and a repeat procedure with the improved second-generation balloon might be a promising strategy. A first advantage concerns safety [5], as only one transseptal puncture is required. In addition, CB ablation is a more straightforward and fast procedure, and outcomes are less heterogeneous [16] and less operator dependent.

A key mechanism of recurrent paroxysmal atrial fibrillation after ablation is pulmonary vein (PV) reconnection, which may reflect lack of efficacy in achieving transmurality and long-lasting lesions [7]. It has been suggested that with CB procedures, the (intensity of) contact of the balloon catheter may not be equally good in all parts of the PV ostium, appreciating the high degree of variability in anatomy and the uniform design of the balloon [10, 17]. This was the rationale of the so-called double-balloon strategy, performed during index procedures for persistent AF. With use of two different balloon sizes during one procedure, a success rate of 70% at 1 year was observed [17].

This strategy aimed to achieve optimal anatomical PV ablation, due to anatomical contact at a different level of the pulmonary vein ostium (either more ostial, 23 mm, or more antral, 28 mm). In addition to PVI, this approach would provide additional substrate modification [18]. Finally, this double-balloon ablation, with either more focus towards the ostium, or towards the antrum, may provide optimal coverage of the area of the ganglionated plexi, with the majority located in close proximity of the PV-LA junction [19–28].

In the abovementioned context, we systematically performed each redo procedure with a different balloon size as compared to the first procedure. As for procedural characteristics, we describe the proportion of pulmonary veins with chronic PV isolation and the total number of reconnected PVs per patient. With regard to clinical outcome, we report safety and freedom of AF at 12 and 24 months.

## Methods

### Patient cohort

Patients underwent a redo CB ablation for ECG and/or Holter-documented recurrences of paroxysmal AF after an initial CB procedure with successful PVI. Paroxysmal AF was defined as episodes of irregular atrial arrhythmia lasting longer than 30 s and terminating within 7 days (spontaneously or with cardioversion). Persistent AF was defined as episodes that last longer than 7 days [1].

To ensure a minimal follow-up evaluation of 1 year, we studied all redo procedures performed with the CB-2 between October 2012 and November 2016 at the Radboudumc, Nijmegen, The Netherlands. Patients younger than 18 years were not eligible for ablation of AF at our institution, neither were patients with an estimated life expectancy < 1 year. Patients with severe comorbidity, left ventricular ejection fraction < 35%, or more than grade 2 valvular disease were not eligible for the staged variable balloon size protocol. Given the observational design of the study, written informed consent was not necessary to obtain according to the Dutch Act on Medical Research involving Human Subjects.

### Periprocedural management

A computed tomography (CT) scan was performed for three-dimensional anatomy of the PVs. Anatomical variants (i.e., common ostium) were also eligible to undergo CB procedures. All patients used oral anticoagulation therapy prior to the procedure.

In case of vitamin K antagonists (VKAs), an uninterrupted anticoagulation strategy was followed with an international normalized ratio (INR) target range of 2.0–3.0 [29]. In case of non-vitamin K antagonist oral anticoagulants (NOACs), we adopted an interrupted regimen, with a pre-procedural NOAC-free interval of 12–24 h. Oral anticoagulation was continued for 3 months, after which life-long treatment was based on the CHA_2_DS_2_-VASc score. Antiarrhythmic drugs were continued for at least 3 months (blanking period) after the procedure.

### First ablation

Procedures were performed under general anesthesia or conscious sedation, with transesophageal echocardiography (TEE) to exclude thrombi in the left atrium. After two punctures in the right femoral vein, a steerable decapolar diagnostic catheter was positioned in the coronary sinus (CS). Access to the left atrium and PVs was obtained by performing a single transseptal puncture using the 8,5-French (SL0, St. Jude Medical) under fluoroscopic guidance and TEE imaging. Directly thereafter, an initial bolus of 70 IU/kg heparin was administered, and the ACT was determined at 20-min intervals to ensure a target activated clotting time (ACT) > 300 s.

All ablations were performed with a CB (Medtronic, Minneapolis, MN, USA) in combination with the 12-French steerable sheath (Medtronic, Minneapolis, MN, USA). During the first procedure, isolation of the PVs with disappearance of real-time potentials was confirmed by absence of reconnection after adenosine. All the PVs were mapped with an inner lumen mapping catheter (ILMC) (Achieve Mapping catheter 20 mm Arctic Front, Medtronic, Minneapolis, MN, USA). Vessel occlusion was considered optimal when selective contrast injection showed total contrast retention. Every PV was treated with at least two applications. The freeze cycles were 240 s for CB-1 and 180 s for CB-2. Balloon size of the CB was either 23 or 28 mm. In case of an RSPV diameter > 21 mm at CT imaging, we opted for a 28-mm balloon during the index procedure to reduce the risk of phrenic nerve (PN) palsy [30]. To early detect PN palsy, PN pacing was performed during applications on the right-sided veins, with the decapolar catheter from the CS positioned in the superior vena cava (SVC). Before the first application, capture was determined with palpation on the abdomen of the hemidiaphragmatic excursion. The PN pacing was started when the balloon temperature reached − 30 °C (20 mA at 1.0 ms pulse width at a cycle length of 1200 ms). When diminished diaphragm movements were noticed, the ablation was interrupted with immediate balloon deflation [31].

The day after the procedure, a protocol-driven transthoracic echocardiogram (TTE) was performed to exclude pericardial effusion after which the patient was discharged.

### Redo procedure

All redo procedures were performed with CB-2, and the setup of the second ablation procedure was similar to the initial procedure except for the size of the balloon. Patients with a 23-mm balloon in the first procedure underwent their redo procedure with a 28-mm balloon and vice versa.

In general, this particular redo strategy ensures a different anatomical match between the balloon and the ostium of the pulmonary vein than during the index procedure, which may have two advantages. First, it allows for pulmonary vein isolation at a different level, either more ostial (23 mm) or more antral (28 mm) than during the index procedure. In fact, despite optimal occlusion during the index procedure, the circumferential intensity of the lesion may not have been equal in all parts of the vein due to anatomical reasons, which may have contributed to reconnection. This was the key argument for the variable balloon size strategy.

A second advantage is that the use of two different balloon sizes may contribute to a more comprehensive anatomical ablation. The 28-mm balloon would also provide additional antrum modification, which has been implicated as part of the success of cryoballoon ablation [18]. On the other hand, on top of PVI at a different level, a 23 mm balloon was expected to provide a higher intensity ablation at the level of the ostium, which may have better impact on the GPs given their preferential localization at the PV LA junction [19].

When mapping of the PV showed reconnection, two applications were performed. In the absence of reconnection our protocol specified a single freeze, appreciating the role of fractionated ostial potentials as a contributor to recurrences [9, 32], and the abovementioned impact of additional substrate ablation and GP modification [18, 25]. An additional argument was that, reconnection may have gone undetected in some cases, as there was no systematic administration of adenosine prior to the redo. Moreover, despite the good correlation between the ILMC (Achieve) and the Lasso, there is no flawless 1:1 correspondence [33].

### Follow-up and endpoint

Follow-up was systematically collected and included (1) protocol-driven, intensive Holter monitoring, (2) systematic outpatient follow-up, and (3) symptom-driven clinical follow-up. Holter follow-up comprised 6-day Holter monitoring at 6 and 12 weeks, and at 6 and 12 months after ablation. Successive visits at the outpatient clinic were scheduled 1–2 weeks after each of the respective Holter recordings, and each visit included a resting ECG and physical examination. In addition, all patients were encouraged to contact the hospital in case of palpitations to incorporate ECG-documented recurrences observed during ER visits and admissions.

As endpoints of this study, we assessed the proportion of patients without AF recurrence at 12 and 24 months, appreciating a blanking period of 3 months after the procedure.

### Statistical analyses

Categorical variables are presented as proportions, and continuous variables are presented as means ± standard deviations (SD) or medians with interquartile ranges.

Comparisons between categorical variables were made with chi-squared tests or Fisher’s exact tests. Furthermore, we used Kaplan-Meier analyses for the estimation of AF recurrence at 2 years of follow-up. Potential risk factors for AF recurrence on univariable analyses (*p* < 0.10) were assessed in a multivariable model, using binary logistic regression. A two-tailed *p* value < 0.05 was considered statistically significant. We used SPSS version 22.0 (SPSS, Inc., Chicago, IL, USA).

## Results

### Study group and baseline characteristics

**Fig. 1:**
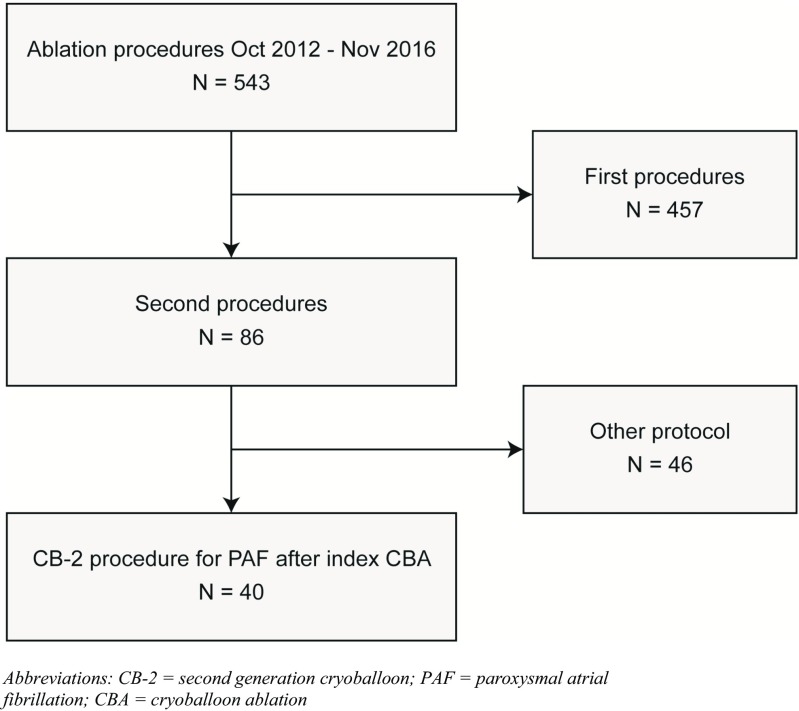
Flowchart of study population

During the study period, there were 543 ablations which included 86 redo procedures (16%). Of these, 40 were CB-2 procedures for recurrent paroxysmal AF using the variable balloon size strategy (Fig. 1). Mean age of the study population was 59 years (± 9.5 years) and 22 (55%) were male. Patients had a median CHA_2_DS_2_-VASc score of 1 (IQR 0–3) and a median left atrial volume index of 33 mL/m^2^ (IQR 24–37). The index procedure was performed with a CB-1 in 16 patients (40%), and a 28 mm balloon was used in 31 patients (78%) during this initial procedure. Median time between the initial and redo procedure was 13 months (IQR 7–23). In anticipation of the redo procedure, 53% (*n* = 21) of patients were on class I or III antiarrhythmic drugs (Table 1).

**Table 1:** Baseline demographic and clinical characteristics

Baseline characteristic	Study patients (*n* = 40)
Age in years, mean (± SD)	58.7 (9.5)
Male gender	22 (55%)
Body mass index in kg/m^2^, median (IQR)	26.3 (23.6–29.4)
Months since AF diagnosis, median (IQR)	62 (31–127)
Number of cardioversions, median (IQR)	1 (0–3)
CHA_2_DS_2_-VASc score, median (IQR)	1 (0–3)
Hypertension	21 (53%)
Coronary artery disease	0 (0%)
Diabetes mellitus	1 (3%)
Previous stroke/transient ischemic attack	1 (3%)
Peripheral artery disease	1 (3%)
Glomerular filtration rate < 60 mL/min	1 (3%)
Left ventricular ejection fraction in %, median (IQR)	61 (60–61)
Left atrial volume index ≥ 34 mL/m^2^	18 (45%)
Months since first procedure, median (IQR)	13 (7–23)
Balloon generation at index procedure	
First-generation balloon	16 (40%)
Second-generation balloon	24 (60%)
Balloon size at index procedure	
23 mm balloon	9 (23%)
28 mm balloon	31 (78%)
Oral anticoagulation therapy	
Vitamin K antagonist	30 (75%)
Non-vitamin K oral anticoagulant	10 (25%)
Current use of antiarrhythmic drugs	
Class I agent	12 (30%)
Class III agent	9 (23%)
Previously used antiarrhythmic drugs	
Class I agent	13 (33%)
Class III agent	14 (35%)

*SD* standard deviation, *IQR* interquartile range, *AF* atrial fibrillation

### Procedural information

The median procedure time was 65 min (IQR 60–85 min) with a median fluoroscopy time of 18 min (IQR 12–21 min), as displayed in Table 2. After gaining access to the left atrium, all PVs were first mapped using the ILMC (Achieve).

**Table 2:** Procedural characteristics of repeated catheter ablations

Procedural characteristic	Study patients (*n* = 40)
Procedural time in minutes, median (IQR)	65 (60–85)
Fluoroscopy time in minutes, median (IQR)	18 (12–21)
Number of reconnected pulmonary veins	
0	12 (30%)
1	19 (48%)
2	6 (15%)
3	3 (8%)
4	0 (0%)
Reconnected vein	
Left superior pulmonary vein	18 (45%)
Left inferior pulmonary vein	8 (20%)
Right superior pulmonary vein	14 (35%)
Right inferior pulmonary vein	0 (0%)

*IQR* interquartile range

Durable electrical PVI was observed in 120 of 160 veins (75%). Among the patients with CB-1 in the initial procedure, the proportion of chronic PVI was 41/64 (64%), in contrast to 79/96 (82%) in patients with a CB-2 used in the initial procedure (*p* = 0.01). The corresponding numbers of reconnected veins per patient were 1.0 (40/40) in the entire cohort, 1.4 for patients with an initial CB-1 procedure and 0.7 for patients with an initial CB-2 procedure (*p* = 0.008).

In total, 48 (60%) superior PVs were isolated, as compared to 72 (90%) of the inferior PVs (*p* < 0.001). When comparing the left veins (LSPV and LIPV) and the right veins (RSPV and RIPV), a total of 54 veins (68%) were isolated at the left side and 66 (83%) at the right side (*p* = 0.04). The numbers of individual isolated veins were 22 (55%) for the LSPV, 32 (80%) in the LIPV, 26 (65%) in the RSPV, and 40 (100%) in the RIPV.

In total, 12 patients (30%) showed durable isolation in all veins. Importantly, after CB-1 ablation in two patients (13%), all PVs appeared isolated, as compared to ten patients (42%) after an initial CB-2 procedure (*p* = 0.08).

### Long-term success rates

All patients had a minimal follow-up of 12 months, and median follow-up was 17 months (IQR 14–39). At 1 year, 28 patients (70%) were free of AF recurrence (Table 3). Among the 12 patients with AF recurrence, 10 (83%) were detected as part of a clinical presentation at the outpatient clinic or chest pain unit with documented AF. In three cases, recurrent palpitations were documented as AF by protocol-driven Holter monitoring. AF-free survival at 2 years was 56%. In the multivariable analysis, only the number of previous electrical cardioversions was independently associated with AF recurrence at 1 year (hazard ratio 1.47, 95% confidence interval 1.03–2.10).

**Table 3:** Recurrence of atrial fibrillation at 1 and 2 years after redo ablation

1 - year recurrence-free survival	70%^a^ (28)
Documented recurrence	30% (12)
ECG with clinical presentation	25% (10)
Holter registration	8% ^b^ (3)
2 - year recurrence-free survival	56%^c^

^a^All patients had at least one year follow-up

^b^In one case, detection both by routine Holter monitoring and by clinical presentation at the Chest Pain Unit

^c^Event rates by Kaplan Meier, median follow-up was 17 months (IQR 14-39)

### Complications

No major periprocedural complications (thromboembolic events, tamponades, major bleedings, atrioesophageal fistulas) occurred. No complications due to the vascular access were noticed. Transient PN palsy was seen in seven patients (18%) during the redo ablation, of which two patients were treated with a 23 mm CB during the redo procedure. Immediate balloon deflation was done when PN palsy was noticed. Despite the interrupted freeze, all the PVs were proven isolated. All PN palsies recovered before the end of the procedure.

## Discussion

To our knowledge, this is the first report on the efficacy and safety of a systematic strategy of repeat procedures with a CB-2 after an initially successful PVI with CB ablation. With 1- and 2-year success rates of 70 and 56%, respectively, outcome is acceptable and procedural safety was good. We identified the number of previous cardioversions as a predictor of AF recurrence.

### Redo strategies—rationale and evidence

Information on the optimal technique for a repeat procedure for recurrent AF is scarce and approaches vary, as do the reported success rates [1, 2, 6–12]. As of yet, the experience and available evidence for redo procedures has been in favor of RF ablation. A randomized comparison between CB-1 and RF as repeat procedure after an index RF procedure resulted in markedly better AF-free survival after repeat RF [11]. Appreciating the 40–60% recurrence rates after a redo, several groups have studied an approach of more extensive ablation and suggested better outcomes, but randomized evidence does not corroborate [1, 2, 15].

With the introduction of the CB-2, rates of up to 75–90% durable PVI have been reported [34], significantly better than observed with the CB-1 [35], and also higher than with RF ablation [7, 36]. Recently, a first series of CB-2 redo procedures has been described after an index RF procedure, with reported rates of durable PVI of 54% at the time of the redo, and a 1-year success rate of 83% [12]. These observations support further study on CB-2 ablations as a repeat procedure, as it could be a promising safe [5], straightforward, and fast strategy, with more reproducibility in outcomes than RF ablation [16].

In follow-up on the rationale of previous work, we defined a redo protocol with the staged use of two different balloon sizes as we hypothesized that this would allow for different (intensity of) contact during pulmonary vein occlusion. Appreciating there was total pulmonary vein occlusion and successful isolation during the index procedure, we now aimed for pulmonary vein isolation at a different level, either more ostial (23 mm) or more antral (28 mm). This was the key argument to use a larger, or smaller, balloon during the redo.

Procedures performed with the 28 mm balloon were expected to result in additional substrate modification, which could be of added value, in terms of posterior wall antrum ablation and potentially targeting rotors and non-PV triggers [18, 37–39].

Finally, with this redo approach, we aimed to achieve a more comprehensive anatomical ablation of the area where the (networks to the) GPs are located [19], with the intention to focus the ablation more towards the ostium (23 mm) or the antral area (28 mm) of the pulmonary vein. As initiation and maintenance of AF is also affected by GPs [20–24, 28], this approach may provide additional benefit for AF-free survival [25–27]. Appreciating the abovementioned rationale and the higher force of CB-2 balloons, we adopted this systematic approach of redo procedures with a CB-2 balloon using a different balloon size as compared to the index CB procedure.

### Repeat procedure—durable PV isolation

Our overall proportion of 75% chronic PVI was a composite of 64 and 82% for first- and second-generation cryoballoon index procedures, respectively, which confirms the high durable isolation with the latter, with rates comparable to previous reports [7–10, 13, 32, 34–36]. Remarkably, in our series, the right inferior PV had the highest rates of durable isolation, which contrasts previous observations [7, 9, 32, 36]. Durable isolation in all PVs was observed in about a third of patients, in line with previous reports. Notably, in the absence of reconnection, fractionated ostial potentials have been implicated as a contributor to recurrences, with several groups performing additional ablation in the setting of a redo [9, 32]. Moreover, the Achieve catheter has a good but not flawless 1:1 correspondence with the Lasso catheter and electrical activity may have gone undetected in some cases [33]. Finally, there was no systematic administration of adenosine at the start of the repeat procedure.

In the aforementioned context, and appreciating the fact that all patients had the clinical presentation of recurrent paroxysmal AF, our protocol specified a single freeze in PVs that showed no signs of reconnection. Interestingly, complete isolation at the start of the repeat procedure was not a predictor of higher rates of AF recurrence, which may be an indirect clue that also other mechanisms than pulmonary vein isolation may be implicated in the process of recurrences.

### Repeat procedure—AF-free survival

Considering the outcomes after an index ablation procedure [5, 6], the observed 1- and 2-year success rates of 70 and 56% are very acceptable for repeat procedures and are higher than the rates reported for redo procedures with a CB-1 after an index CB ablation [13]. Similar considerations hold true for the AF-free survival rates achieved with a CB-2 procedure after an index RF ablation [12]. The markedly better acute and long-term durability of PV isolation with the CB-2 may be one of the explanations for the improved rates we observed.

At present, the majority of evidence on outcome after repeat procedures pertains to RF procedures. Reported success rates of RF redo procedures after an index CB-1 are 86% at 6 months and 73% at 2 years [8, 10]. As for index procedures with a CB-2, a repeat procedure with RF has been reported to result in 4–6-month success rates of 69–89% and a 1-year result of 83% [7–9].

### Implications

Our observations of 82% chronic PV isolation with the CB-2, and the acceptable success rates after the repeat procedure provide a first indication of the potential to perform redo procedures for (paroxysmal) AF with the second generation cryoballoon. Due to the variety in reported approaches, study populations, and chosen time points to report outcomes, caution is warranted with regard to comparative conclusions with RF, which calls for head to head comparisons.

Whereas the present protocol was rather straightforward, a systematic approach with adenosine, Lasso measurements, and more detailed mechanistic studies could have provided more insight into to underlying mechanisms of recurrence. More detailed studies are warranted to optimize (longer-term) outcomes after repeat procedures, and assess whether and how the focus should shift towards approaches focused on additional substrate modification and/or ganglionated plexi ablation on top of PVI [1, 2].

### Limitations

Although this represents the first systematic cohort of second generation cryoablation redo procedures after an index cryoballoon ablation, our sample size is limited and results only pertain to patients with recurrent paroxysmal atrial fibrillation treated in a medium-volume center with experienced operators [40]. In lack of a control group, it is uncertain how much the use of a different balloon size during the second procedure contributed to the outcomes. Before implementation on a larger scale, our findings require confirmation.

## Conclusions

This strategy of repeat ablation with a second-generation cryoballoon, using a staged variable balloon size approach, is safe and results in acceptable outcomes at 1 and 2 year follow-up.

With the high durable rates of chronic pulmonary vein isolation achieved with the second generation cryoballoon, randomized studies on the optimal technique for repeat procedures are eagerly awaited.
